# A Community and Hospital cAre Bundle to improve the medical treatment of severe cLaudIcation and critical limb iSchaemia (CHABLIS)

**DOI:** 10.3310/nihropenres.13341.1

**Published:** 2022-11-28

**Authors:** Emma Watson, Bernadeta Bridgwood, Prakash Saha, Matthew Bown, Ruth Benson, Vanessa Lawrence, Clair Le Boutillier, Daniel Lasserson, Sarah Messeder, Athanasios Saratzis

**Affiliations:** 1 Cardiovascular Sciences, University of Leicester, Glenfield Hospital, Leicester, Leicestershire, LE39QP, UK; 2 Cardiovascular Sciences, Vascular Surgery, King's College London SE1 1UL, London, UK; 3 Department of Surgery, University of Birmingham, UK, Birmingham, UK; 4 Institute of Psychiatry, Psychology & Neuroscience, King's College London SE1 1UL, London, London, UK; 5 Health Sciences, University of Warwick, Warwick, UK

**Keywords:** Peripheral Artery Disease (PAD), best medical therapy, claudication, chronic limb threatening ischaemia

## Abstract

**Background:**

Patients with peripheral artery disease (PAD) often do not receive optimal best medical therapy (BMT). Through interaction with patients and healthcare-professionals (HCPs) we developed the LEaflet Gp letter Structured checklist (LEGS) complex clinical intervention to support HCPs in providing guideline-compliant PAD BMT.

**Methods:**

This was a prospective multicentre study assessing the feasibility and fidelity of delivering the LEGS intervention in primary and secondary care over six months. Intervention fidelity was scored based on the proportion of intervention components used correctly at discharge, 30 days, and six months.

**Results:**

Overall, 129 individuals were screened and 120 took part (33% female, 74% with chronic limb threatening ischaemia; 93% recruitment rate). Of those, 118 (98% retention rate) completed follow-up. Mean intervention fidelity score at discharge (primary outcome measure) was 63% [95% Confidence Interval (CI): 39-68%, SD: 5%], exceeding the success criteria set at 60% by a panel of HCPs and patients. This, however, declined to 51% at six months. Eight patients (6.7%) died (all cardiovascular deaths), four (3.3%) had a major lower limb amputation, 12 (10%) had a cardiovascular event, and 13 (11%) were admitted due to limb ischaemia at six months. Incomplete lipid therapy prescriptions and LEGS intervention documents not received by primary care CHPs were the most common reasons for not complying with the LEGS intervention.

**Conclusions:**

The LEGS intervention can be delivered in PAD care pathways across different hospitals, primary, and community healthcare settings with acceptable fidelity, to streamline and improve PAD BMT short- and medium-term.

## Introduction

Peripheral Artery Disease (PAD) affects a fifth of people over 60 years ^1– 3^. It is the commonest cause of lower limb amputation and leading cause of cardiovascular morbidity ^4^. Patients with PAD who develop symptoms typically present either with intermittent claudication (IC) or chronic limb threatening ischaemia (CLTI). More than half of those diagnosed with symptomatic PAD are expected to die, have an amputation or a major cardiovascular event within five years ^2, 5– 9^. Many of their cardiovascular risk-factors are modifiable ^9^. Current international guidance sets clear targets regarding how these risk factors should be addressed to prevent cardiac events and amputations ^3, 10^. Controlling these modifiable risk factors would lead to a 29% absolute risk reduction of their median ten-year cardiovascular, adding six cardiovascular disease-free years to their life expectancy ^9^. Previous work, however, has shown very poor implementation of this guidance in routine practice ^9^. Only a tenth of patients are prescribed appropriate preventive medication once they have developed symptoms owing to PAS. The vast majority continue to have high blood pressure or smoke and almost none receive structured lifestyle or dietary support ^9^ after being diagnosed with symptomatic PAD.

In an attempt to address this healthcare issue, we conducted patient and public involvement (PPI) workshops and interviews with patients, primary and secondary care professional stakeholders to identify key barriers in implementing guidance in routine clinical care when treating patients with PAD. Based on these, we developed a bundle of documents (checklists and letters) for use in both primary and secondary care, the “LEGS” (LEaflet Gp letter Structured checklist) intervention in order to improve the medical care of patients with symptomatic PAD across primary and secondary care. LEGS is a PAD-focussed complex clinical intervention that is grounded in the needs and perceptions of patients with PAD, the views of primary and secondary care clinicians, behavioural change theory, as well as latest national and international guidance ^10, 11^.

The aim of this multicentre prospective cohort study was to test whether LEGS can indeed be used in current PAD care pathways across secondary/primary care at multiple different institutions and regions.

## Methods

### Study design and regulatory approvals

The “Community and Hospital cAre Bundle to improve the medical treatment of cLaudIcation and critical limb iSchaemia” (CHABLIS) study was a prospective multicentre mixed methods study, delivered in four National Health Service (NHS) regions between September 2019 to 2021. The research was reviewed and approved by the NHS Wales Research Ethics Committee (reference 20/WA/0319) and NHS Health Research Authority (HRA). It was funded by the National Institute for Health Research NIHR Research for Patient Benefit (RfPB) programme (NIHR202008). It was prospectively registered on an online repository; the protocol and LEGS intervention constituents have been made available online (reference: ISRCTN13202085, free to access and use). The study was sponsored by the University of Leicester. Patients provided written informed consent before taking part. The funder had no input in study conduct or analyses.

### Aim and objectives

The main aim was to assess the fidelity and feasibility of delivering the LEGS complex clinical intervention as part of the care of patients with symptomatic PAD referred to secondary care.

The first objective was to quantitatively assess the fidelity of delivering LEGS. The secondary objectives included assessing recruitment and retention rate to receiving PAD care based on the LEGS intervention. A formal qualitative assessment of the perceived acceptability of LEGS is presented separately ^12^.

***Study population (inclusion criteria)***

Individuals >18 years of age with severe IC or CLTI (Rutherford stages 3–6) referred to secondary care. Recruitment lasted six months (March 2021-September 2021) across:

-  Leicester Vascular Institute,

-  Guy’s & St Thomas’ NHS Foundation Trust,

-  University Hospitals Birmingham NHS Trust,

-  University Hospital of Coventry & Warwickshire NHS Trust.

Patients were recruited from inpatient wards on the day of admission and all outpatient clinics. The recruiting sites include patients from urban and rural backgrounds, providing vascular services 8.2 million.

### Outcome measures

Primary outcome measure: Fidelity of LEGS intervention delivery.

Secondary outcome measures: Proportion of patients agreeing to take part out of those invited (intervention recruitment rate); proportion who provide data at end of follow-up (intervention retention rate); cardiovascular events; mortality; Body Mass Index (BMI); self-reported medication adherence [using the Brief Adherence Rating Scale ^13^]; smoking status (self-reported); uptake of smoking-cessation services for smokers; cholesterol, low-/high-density lipoprotein levels.

### Development of the LEGS intervention

**Figure 1.**
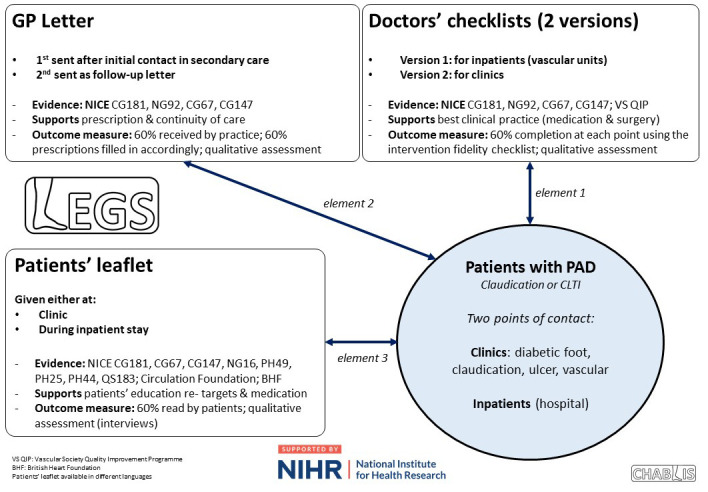
Infographic detailing each element of the complex clinical intervention, which healthcare provider receives the relevant element, literature/evidence, and success criteria (outcome measures) for this study.

LEGS was developed prior to this study ( Figure 1) based on co-creation principles ^14^. A review of best available evidence and PAD guidelines identified 5 key treatment areas [PAD best medical therapy (BMT)] ^3, 10, 15^: Lipid control; antiplatelet therapy; Blood pressure (BP) control; smoking cessation; glucose control.

Following that, four workshops were held, involving 91 patients with CLTI, 24 with IC, four carers, 17 nurses, four general practitioners (GPs), and five surgeons, to examine barriers and facilitators of delivering BMT; based on these, a set of documents (printed and digital format) were created to address barriers and facilitate BMT.

### LEGS intervention constituents

***Inpatient healthcare professionals’ checklist***

One-page checklist for patients admitted in a hospital setting. The list was checked initially with the first patient clerking (on admission) by the relevant HCP and then immediately before discharge.

***Outpatient doctors’ checklist***

One-page checklist for those seen in a clinic. It was developed with the help of clinic co-ordinators, GPs, nurses, and surgeons. It was used during consultation prompting the HCP to address all 5 PAD BMT areas.

***Leaflet for patients and relatives***

A lay leaflet with information aiming to support patients achieve BMT targets. The British Heart Foundation and Circulation Foundation PAD documents were used as a basis on which the patients’ helping us with the development pathway expanded as per their perceptions/preferences and most current BMT guidance ^2, 10^. This was given to the patient upon presentation and mailed to the GP (available in five languages).

***GP letter***

A standardised letter with action points relating to BMT. This was developed by GPs in 11 different regions. The letter was sent to the GP after discharge and each clinic visit. This addressed the poor and confusing secondary care communications.

***GP follow-up letter***

A follow-up letter sent automatically to the GP four weeks after each contact with secondary care prompting action for BMT targets.

The intervention schematic and protocol (see Extended data) are available online (digital) free-of-charge.

### Definitions and reporting

Definitions were as per the reporting standards of the Society for Vascular Surgery for PAD ^10^. Reporting followed the “Strengthening the reporting of observational studies in epidemiology” guidance.

### Follow-up procedures

-  Baseline; collection of clinical presentation information and cardiovascular risk-factors.

-  Assessment 1; day of discharge or clinic appointment. The number of the LEGS intervention components filled in correctly were recorded the.

-  Assessment 2; 15 days discharge or clinic appointment. The GP was contacted to assess whether they received and read the LEGS letter and followed advice regarding BMT.

-  Assessments 3 and 4; 30 days and six months. Participants were followed-up in person. Patients’ notes were reviewed alongside routine biochemistry performed at each visit. Prescriptions were obtained from patients and GPs. 

## Fidelity assessment

A purpose-built intervention fidelity (IF) checklist was used to assess the presence or absence of each intervention element when providing care for these patients compared to the instructions of the LEGS intervention. The proportion of the LEGS elements that were delivered appropriately were then reported per patient at baseline (first contact with secondary care), discharge, and six months. We also assessed whether the primary care letters had been received by the patient’s GP two, six weeks, and six months after discharge.

### Sample size calculations and statistical analysis

Based on our pre-study work with stakeholders (focus groups and surveys), we were willing to accept the LEGS intervention as standard of care in clinical PAD pathways if 60% of the components were delivered as intended within 15 days of follow-up, whilst accepting a 10% margin of error. A total of 93 patients had to complete the follow-up in order to report this within a 95% confidence interval with 90% power.

Analyses were performed using SPSS version 24.0 and R software. A pre-drawn statistical analysis plan was formulated and included in the study protocol. Data are presented as mean with standard deviation (SD) or absolute values with proportions. Normality of distributions was assessed using the Kolmogorov-Smirnov test and the degree of skewness and kurtosis of each variable of interest. Comparisons were performed using either the t-test, Mann-Whitney U or Wilcoxon Test, or Fisher’s exact test for comparisons between two groups, based on the nature of the data compared. For comparisons across groups, Analysis of Variance (ANOVA) was used. A p value <0.05 was considered statistically significant. Data was maintained and analysed by the NIHR Leicester Biomedical Research Centre (BRC), independent to investigators.

## Results

### Baseline characteristics

Overall, 120 participants (40 female, 33%) took part. Table 1 describes baseline characteristics (upon referral to secondary care specialist vascular services). Of interest, 89 patients (74%) presented with CLTI (19 with tissue loss, 16%). Overall, 40 (33%) were inpatients at baseline and 80 (67%) were seen in a clinic. One was seen in clinic and admitted to the ward on the same day (to undergo a major amputation due to fulminant sepsis; Rutherford stage 6). Table 2 describes the types of vascular procedures/interventions that 84 patients underwent after their index appointment. All claudicants were initially treated with conservative means. Overall, 129 patients were approached to take part, of which 120 consented (93% intervention recruitment rate). A total of 118 (98% intervention retention rate) completed six-month follow-up i.e. their care was provided based on the LEGS intervention for at least six months. The mean intervention fidelity score at baseline ( *i.e.* day of the clinic appointment or day of discharge for inpatients - primary outcome measure) was 63% [95% Confidence Interval (CI): 39-68%, SD: 5%]. Table 3 describes the intervention fidelity scores at baseline, 30 days, and six months (end of prospective follow-up) for inpatients and outpatients. At 15 days after taking part in the study, the GPs of 108 patients (90%) had received the LEGS intervention GP leaflets and letters and 119 patients (99%) had received the LEGS education document. Of these, 81 (68%) had read and understood the document; only four asked for a translated version (3%). Of the 81 who read the LEGS leaflet, 66 (81%) needed help from carers or healthcare staff and/or had queries. During six months of follow-up, eight patients (6.7%) died (all cardiovascular deaths), four (3.3%) had a major lower limb amputation, 12 (10%) had a major cardiovascular event other than re-admission for a lower-limb pathology, 12 (10%) were admitted due to CLTI, one (1%) was re-admitted due to acute lower limb ischaemia and underwent a surgical embolectomy (previous superficial femoral artery angioplasty), and 12 (10%) underwent re-intervention. Overall, 43 (36%) saw their GP at least once after they took part in the study, and 22 (18%) had at least one community care appointment. Table 4 describes pharmacotherapy prescribed before the patients took part in the study, at baseline, and follow-up. More patients were prescribed an antiplatelet, statin, an anticoagulant, and blood pressure medications at six months compared to baseline.

**Table 1.** Baseline characteristics per patient.

**Age (years)**	71 (SD: 10)
**Male**	80 (67%)
**Intermittent Claudication (all ** **Rutherford 3)**	31 (26%)
**CLTI**	89 (74%)
**Rest pain**	80 (67%)
**Tissue loss**	19 (16%)
**Tissue loss - Rutherford stage 6**	9 (7.5%)
**Hypertension**	110 (92%)
**Hypercholesterolemia**	78 (65%)
**Hyperlipidaemia**	73 (62%)
**Previous MI**	19 (16%)
**Previous TIA**	5 (4%)
**Previous stroke**	5 (4%)
**Diabetes**	96 (80%)
**Heart failure**	5 (4%)
**AF**	64 (53%)
**CKD**	79 (66%)
**Dialysis**	5 (4.2%)
**Current smoker**	67 (56%)
**Ex-smoker**	32 (26%)
**Ezetimibe**	3 (2.55%)
**Statin**	53 (44%)
**Aspirin**	43 (36%)
**Clopidogrel**	44 (37%)
**Ticagrelor**	1 (1%)
**Prasugrel**	1 (1%)
**Warfarin**	5 (4%)
**NOAC**	22 (18%)
**Previous angioplasty**	11 (9%)
**Previous open or hybrid ** **revascularisation**	2 (2%)
**Total cholesterol**	6.1 mmol/l (SD: 1.6)
**HDL**	1.3 mmol/l (SD: 0.8)
**LDL**	3.7 mmol/l (SD: 0.9)
**Anaemia**	26 (22%)
**ABPI**	0.71 (SD: 0.3)
**Toe pressure**	42mmHg (SD: 11)
**WiFi score**	4 (range: 1-5) **

CLTI: Chronic limb-threatening ischaemia, MI: myocardial infarction, TIA: Transient ischaemic attack, AF: atrial fibrillation, CKD: chronic kidney disease, NOAC: novel oral anticoagulants, ABPI: ankle brachial pressure index. Normally distributed variables presented as mean (SD: standard deviation) and categorical data distributed as count (percentage) **WiFi score presented as median and range

**Table 2.** Procedures patients underwent after their index secondary care appointment (at outpatients or inpatient admission) and participation in the study; all procedures performed within 2 weeks of consenting to take part (as part of the patients’ care plan).

Procedure	
Foot debridement	14 (12%)
Minor amputation	14 (12%)
Major amputation (primary treatment due to sepsis)	1 (0.8%)
Angioplasty - infra-inguinal	22 (18%)
Iliac angioplasty & stenting	54 (45%)
Common femoral endarterectomy	2 (1.6%)
Femoro-popliteal bypass	2 (1.6%)
Hybrid revascularisation	48 (40%)
Conservative management (no intervention)	32 (27%)

**Table 3.** LEGS (LEaflet Gp letter Structured checklist) complex intervention fidelity scores at baseline, 30 days, and 6 months (end of prospective follow-up).

Inpatients (admitted on ward at baseline) - 40 participants			
Time point	Discharge day	30 days	6 months
LEGS fidelity score	54%	52%	50%
Outpatients (seen in clinic at baseline) - 80 participants			
Time point	Discharge day	30 days	6 months
LEGS fidelity score	75%	54%	53%

**Table 4.** Pharmacotherapy prescribed per National Institute for Health and Care Excellence (NICE) guidance before the patients took part in the study, at baseline (i.e. once they left clinic or they went home for those who were inpatients) and at 6 months, as well as biochemistry and relevant cardiovascular risk factors.

Inpatients (admitted on ward at baseline) - 40 participants				
Time point	Baseline	Discharge day	30 days	6 months
Antithrombotic therapy	14 (35%)	40 (100%)	27 (68%)	27 (68%)
Lipid therapy	24 (60%)	33 (83%)	14 (35%)	12 (30%)
Antihypertensive therapy	0	33 (83%)	23 (58%)	23 (58%)
Outpatients (seen in clinic at baseline) - 80 participants				
Time point	Baseline	Discharge day	30 days	6 months
Antithrombotic therapy	62 (68%)	77 (96%)	77 (96%)	71 (89%)
Lipid therapy	54 (68%)	53 (66%)	51 (64%)	48 (60%)

Table 5 and Table 6 describe secondary outcome measures of interest, including quality of life in detail.

**Table 5.** Secondary outcome measures during follow-up.

Inpatients (admitted on ward at baseline) - 40 participants			
Outcome	Discharge day	30 days	6 months
Major cardiovascular event (not limb-related)	1 (2.5%)	2	12 (30%)
Mortality (all cause) ***	1 (2.5%)	4 (10%)	8 (10%)
Major lower limb amputation	1 (2.5%)	2 (5%)	4 (10%)
Reduction in Body Mass Index (BMI) >= 1 unit	0	0	0
Uptake of smoking cessation services (30 smokers)	0	0	17 (57%)
Medication Brief Adherence Rating Scale >=3	4 (10%)	14 (35%)	0
Systolic blood pressure in mmHg	147 (SD: 12)	150 (SD: 11)	144 (SD: 10)
Diastolic blood pressure in mmHg	82 (SD: 2)	84 (SD: 4)	82 (SD: 3)
HbA1c (%)	5.2 (SD: 0.1)	Not available	5.9 (SD: 0.1)
Outpatients (seen in clinic at baseline) - 80 participants **			
Outcome	Discharge day	30 days	6 months
Major cardiovascular event (not limb-related)	0	0	0
Mortality (all cause)	0	0	0
Reduction in Body Mass Index (BMI) by at least 1 unit	0	0	0
Uptake of smoking cessation services (37 smokers)	0	19 (26%)	19 (51%)
Medication Brief Adherence Rating Scale >=3	31 (39%)	40 (50%)	40 (50%)
Systolic blood pressure in mmHg	142 (SD: 12)	151 (SD: 10)	149 (SD: 10)
Diastolic blood pressure in mmHg	82 (SD: 2)	81 (SD: 3)	81 (SD: 3)
HbA1c (%)	5.8 (SD: 0.1)	Not available	5.6 (SD: 0.1)

SD: Standard Deviation **1 patient seen in clinic was admitted the same day to have an emergency major lower limb amputation due to fulminant sepsis on presentation (reported under those admitted as inpatients) - no further outpatient underwent a major lower limb amputation ***All deaths were secondary to major cardiovascular events.

**Table 6.** Numbers and proportions reporting levels within the EQ-5D dimensions.

	Mobility		Self-care		Usual activities		Pain/discomfort		Anxiety/depression	
Level	Baseline	6 months	Baseline	6 months	Baseline	6 months	Baseline	6 months	Baseline	6 months
**1**	10%	20%	20%	20%	30%	30%	20%	0	10%	0
**2**	34%	24%	24%	24%	14%	34%	24%	66%	34%	50%
**3**	40%	34%	46%	34%	56%	36%	46%	34%	56%	50
**4**	16%	22%	10%	22%	0	-	10%	0	0	0

Quality of life in the 5 areas reported using the EuroQoL tool during follow-up (expressed as proportion of patients who successfully replied to the questions at each follow-up appointment)

## Discussion

This study assessed the feasibility and fidelity of delivering a purpose-built complex clinical intervention (LEGS) as part of providing care for patients with symptomatic PAD across four diverse healthcare regions. Intervention recruitment and retention rates were high (93% and 98% respectively), over a six-month period. The mean intervention fidelity score at baseline was 63%; however, this declined at six months both for inpatients and outpatients to just over 50%. Further, one in ten GPs had not received the LEGS intervention letters or other communication from the hospital within 15 days of the patient having been seen in secondary care.

The high rates of recruitment and retention seen in this study when using the LEGS intervention in routine clinical care reflect the shared optimism that we found among patients, GPs, and secondary care clinicians when we conducted qualitative interviews to assess their satisfaction when using LEGS (presented elsewhere ^12^). Both the qualitative and now quantitative findings of this study suggest that the LEGS intervention could promote a streamlined patient pathway and joined-up working across primary and secondary care in this extremely challenging clinical context ^12^. In the qualitative interviews, participants were hopeful that LEGS could help meet the needs of patients with PAD and address a perceived provision gap in their care. However, some patients also highlighted the importance of tailoring information to individual need and stage of PAD, suggesting that the education leaflet should not be used in isolation but act as a supportive conversation guide that would allow HCPs to address patient priorities and concerns. This tailored model of patient care should be taken into account when scaling up the LEGS intervention across other healthcare environments or countries. It should also be the basis of any future intervention development process in this disease area.

During our preliminary work whilst developing the LEGS intervention in workshops and surveys with multiple lay and expert participants, there was agreement that an intervention fidelity target of 60% would be a reasonable target in terms of defining success at delivering the intervention components in actual clinical practice. This target was met and in fact exceeded at the first appointment in secondary care and up until the point of discharge (75% for clinic appointments and 54% for inpatient admissions). The intervention fidelity scores did decline during follow-up, however, to 53% for those seen as outpatients at baseline and 50% for inpatients. The two main reasons leading to a fall in the fidelity scores both for inpatients and outpatients were: i) GPs not having received the LEGS letters - only 112 of the 240 GP letters had been delivered and read by the sixth month follow-up review; ii) lipid therapy (statin or ezetimibe) not having been recorded on the repeat prescription - only half of the participants still had a repeat prescription for high-dose lipid therapy at the sixth month follow-up review. Besides a tailored approach when introducing the education leaflet to patients, these are the other two main issues on which clinicians must focus when delivering LEGS and treating patients with PAD; in fact, these items might be inter-linked, *i.e.* had the GPs received the LEGS communications at six months, more patients might have been prescribed lipid-lowering therapy at that time. Overall, one of the main areas of focus in the LEGS intervention relates to prescribing the right medications for patients with PAD. In our previous work, we have shown that almost one in two patients with symptomatic PAD in the NHS are not prescribed guideline-compliant BMT even after presentation to secondary care to receive specialised treatment such as revascularisation ^9^. As expected, only 44% of patients taking part in the CHABLIS study, despite them presenting with advanced symptomatology ( Table 1), had been prescribed a statin and only 36% had been given Aspirin on presentation to secondary care. This is a reflection of the study’s pragmatic nature and supports the need of introducing an intervention like LEGS in routine care. These proportions did increase during follow-up; however, lipid therapy prescribing was still nowhere near 100%, especially for inpatients (30% at six months - Table 4). 

### Limitations and next steps

This was a non-randomised prospective study aiming to assess intervention fidelity and feasibility of delivery. This is an essential step in the intervention development pathway set out by the Medical Research Council (MRC) ^11^. Given the non-randomised nature of the research, the cost-effectiveness of the LEGS intervention could not fully be assessed. Further, this research is not powered to examine differences in lipid levels or other surrogate markers of cardiovascular risk before and after the introduction of the LEGS checklist in the care of these patients - this was beyond the scope of the study. This research, however, has shown that the fidelity of the LEGS intervention, especially during the initial period after patients present to secondary care, is acceptable and LEGS can be easily introduced in the routine care of patients with PAD who present with severe IC or CLTI. It has also identified areas on which clinicians will have to focus before further research and when implementing the LEGS intervention (should they wish to do so). The next step before adopting the intervention internationally as essential standard of care is to assess its cost-effectiveness, based on established guidance on complex interventions ^11^. At the same time, all medical targets and constituents of the LEGS intervention are based on level 1A randomised evidence ( *e.g.* treatment for hypertension and hyperlipidaemia) and widely accepted clinical guidelines ^3, 10^. These individual treatment targets and algorithms have been tested in terms of clinical and cost-effectiveness in well-designed large international trials. The LEGS intervention brings together these well-established treatment targets in a streamlined manner, to help HCPs provide guideline-based care. As a result, the individual targets and treatment strategies used in LEGS do not require further assessment in randomised trials; this has already been performed. At the same time, the cost-effectiveness of LEGS is uncertain across different healthcare providers and countries. This must be proven before the intervention becomes a “mandatory” part of PAD care ^11^. In the meantime, LEGS is a safe, cheap, and easily implementable tool which can help clinicians provide streamlined BMT when treating patients with PAD. It is freely available and can be edited/customised for use within regions. 

## Conclusions

The LEGS intervention can be delivered in current PAD care pathways in both primary and secondary care with acceptable fidelity. This study also allowed the identification of areas where the intervention can be refined when used in daily practice, *e.g.* using a tailored approach when introducing PAD education literature to patients and ensuring communication to primary care is received on time. A future randomised study should assess cost-effectiveness of the intervention before it becomes essential standard of care across different regions; however, the intervention can be used by PAD care providers until then with acceptable fidelity, to support them in offering guideline-compliant BMT to patients with PAD, based on their needs.

## Data Availability

The raw data includes sensitive/identifiable information such as date of birth, gender, date of surgical or interventional procedure. Anonymised data are available upon request, which can be submitted to the corresponding author via e-mail ( as875@leicester.ac.uk). Study protocol and intervention documents/schematic are available on ISRCTN registry: https://doi.org/10.1186/ISRCTN13202085
