# Supplementary material for: A Community and Hospital cAre Bundle to improve the medical treatment of severe cLaudIcation and critical limb iSchaemia (CHABLIS)
Source: NIHR Open Res. 2022 Nov 28;2:58. [Version 1] doi: 10.3310/nihropenres.13341.1 (PMC10593312; doi:10.3310/nihropenres.13341.1)
Supplement: Peer review history

# Reviewer report: Reviewer response for version 1

**DOI:** 10.3310/nihropenres.14468.r29128

**Published:** 2023-04-27

**Authors:** Janice Tsui

**Affiliations:** 1 Department of Surgical Biotechnology, Division of Surgery & Interventional Science, Faculty of Medical Sciences, University College London, London, England, UK; 2 Department of Vascular Surgery, Royal Free London NHS Foundation Trust, London, UK

This is well written article on the important area of optimising best medical therapy and risk factor management in patients with symptomatic peripheral artery disease (PAD). 

 The LEGS intervention has been developed with engagement of key stakeholders and the feasibility and fidelity of delivering it through 4 major and varied UK vascular units/ healthcare regions were assessed. 

 The methodology is well considered and described including sample size calculations with clear rationale. 

 I would like to have seen more details regarding the LEGS intervention components within the article.

 The results are clearly presented and important findings are discussed. The low number of symptomatic PAD patients on best medical therapy at baseline is not surprising and highlights the need for interventions such as LEGS. The fidelity target set was realistic and the fact that it was exceeded initially is promising. As the authors point out, it is disappointing and worrying to then see a decline in follow-up. This study allows potential reasons for this to be examined. The limitations of the study are discussed. 

 Do the authors have any thoughts on how to improve the 2 main reasons they suggest led to fall in fidelity scores on follow-up, i.e. GPs not receiving the LEGS letters and the continued prescription of lipid therapy? 

 I agree with the authors that LEGS intervention offers a cheap and practical tool to improve BMT in patients with PAD and look forward to their ongoing work in the area.

Is the work clearly and accurately presented and does it cite the current literature?

Yes

If applicable, is the statistical analysis and its interpretation appropriate?

Yes

Are all the source data underlying the results available to ensure full reproducibility?

Yes

Is the study design appropriate and is the work technically sound?

Yes

Are the conclusions drawn adequately supported by the results?

Yes

Are sufficient details of methods and analysis provided to allow replication by others?

Yes

Reviewer Expertise:

Vascular surgery, PAD,

I confirm that I have read this submission and believe that I have an appropriate level of expertise to confirm that it is of an acceptable scientific standard.

# Reviewer report: Reviewer response for version 1

**DOI:** 10.3310/nihropenres.14468.r28893

**Published:** 2022-12-19

**Authors:** Isabelle Van Herzeele

**Affiliations:** 1 Department of Thoracic and Vascular Surgery, Ghent University Hospital, Ghent, Belgium; 2 Research Foundation-Flanders (FWO), Brussels, Belgium

This prospective multicentre study has evaluated if it is possible to improve the medical treatment of patients with peripheral arterial disease (PAD) e.g. severe claudication and critical limb ischaemia by delivering a LEaflet GP letter Structured checklist (LEGS) to support healthcare professionals to provide guideline-compliant PAD BMT. 

 The LEGS intervention was delivered in primary and secondary care and the intervention fidelity was scored (based on the proportion of intervention components used correctly) at discharge, 30 days, and 6 months. 

 A panel of patients, primary and secondary healthcare professionals had set the success criteria at mean intervention fidelity score of 60% and had created a set of printed and digital documents to address barriers and facilitate BMT in PAD patients.

 - Inpatient one page checklist used with the first patient clerking and immediately before discharge. 

 - Outpatient's doctor's checklist, used during consultation. 

 - A lay leaflet for patients and relatives to support patients in achieving BMT targets. 

 - GP letter with action points relating to BMT, sent after discharge and each clinic visit. 

 - GP FU letter - sent automatically to the GP four weeks after each contact. 

 The CHABLIS study was delivered in four NHS regions between Sept 2019 and 2021. 120 out of 129 patients (89 CLTI- 40 inpatients) consented and 118 pts completed the 6 months follow up. Eight patients died, four had a major lower limb amputation, 12 had cardiovascular events other than lower limb pathology, 12 were admitted due to CLTI, had an acute lower limb ischemia. More patients were described antiplatelets, statins, anticoagulant and blood pressure medication at six months compared to baseline. 

 The LEGS fidelity score was for inpatients - 54%, 52%, 50 % at discharge, 30 days and 6 months respectively; for outpatients 75%, 54%, 53% at discharge, 30 days and 6 months respectively. 90% of the GPs had received the LEGS intervention GP leaflet and letters while 99% of the patients had received the LEGS education document. 81 patients had read the document but 81% needed help from carers or healthcare staff or had queries. 

 This LEGS intervention could promote a streamlined patient pathway and improve collaboration between primary and secondary care in PAD patients. The educational leaflet for the patients should not be used in isolation but be used to support the conversation with the healthcare professionals. 

 The fidelity score was high at discharge but dropped significantly at 6 months. Furthermore the fidelity score was lower with inpatients than with outpatients. This may be due to the fact that GPs did not receive the LEGS letters in about half of the cases at 6 months. This may also explain that BMT, although it had improved, was still not 100% especially not in the inpatient population. 

 Questions to the authors: 

 - This LEGS intervention is ideal to improve to structure the delivery of BMT in patients with PAD who are prone to cardiovascular events and major lower limb problems. Although the results are positive, a lot more work needs to be done to improve the BMT and to control risk factors in this patient population in the UK.

- Would a regular telephone conversation with a healthcare worker improve the adherence of the patients e.g. taking their medication, quit smoking, reduce overweight, healthy diet or pick up any other problems earlier? 
- Does the educational leaflet for patients need to be simplified or are more pictures required to make it more understandable for patients. 
- Why are the results for inpatients (fidelity) less good than for outpatients? Is it related to the severity of the underlying disease, socioeconomic situation of the patients? 

I look forward to future research projects focussing on optimisation of BMT in PAD patients by improving the collaboration between primary and secondary care.

Is the work clearly and accurately presented and does it cite the current literature?

Yes

If applicable, is the statistical analysis and its interpretation appropriate?

I cannot comment. A qualified statistician is required.

Are all the source data underlying the results available to ensure full reproducibility?

Yes

Is the study design appropriate and is the work technically sound?

Yes

Are the conclusions drawn adequately supported by the results?

Yes

Are sufficient details of methods and analysis provided to allow replication by others?

Yes

Reviewer Expertise:

Vascular Surgeon, focussing on carotid, aortic and lower limb pathology.

I confirm that I have read this submission and believe that I have an appropriate level of expertise to confirm that it is of an acceptable scientific standard.
